# Season of Sampling and Season of Birth Influence Serotonin Metabolite Levels in Human Cerebrospinal Fluid

**DOI:** 10.1371/journal.pone.0030497

**Published:** 2012-02-01

**Authors:** Jurjen J. Luykx, Steven C. Bakker, Eef Lentjes, Marco P. M. Boks, Nan van Geloven, Marinus J. C. Eijkemans, Esther Janson, Eric Strengman, Anne M. de Lepper, Herman Westenberg, Kai E. Klopper, Hendrik J. Hoorn, Harry P. M. M. Gelissen, Julian Jordan, Noortje M. Tolenaar, Eric P. A. van Dongen, Bregt Michel, Lucija Abramovic, Steve Horvath, Teus Kappen, Peter Bruins, Peter Keijzers, Paul Borgdorff, Roel A. Ophoff, René S. Kahn

**Affiliations:** 1 Department of Psychiatry, Rudolf Magnus Institute of Neuroscience, University Medical Center Utrecht, Utrecht, The Netherlands; 2 Department of Psychiatry, ZNA hospitals, Stuivenberg, Antwerp, Belgium; 3 Department of Clinical Chemistry and Hematology, University Medical Center Utrecht, Utrecht, The Netherlands; 4 Julius Center for Health Sciences and Primary Care, University Medical Center Utrecht, Utrecht, The Netherlands; 5 Clinical Research Unit, Academic Medical Center, Amsterdam, The Netherlands; 6 Department of Biostatistics, Julius Center for Health Sciences and Primary Care, University Medical Center Utrecht, Utrecht, The Netherlands; 7 Department of Medical Genetics, University Medical Center Utrecht, Utrecht, The Netherlands; 8 Department of Human Genetics, David Geffen School of Medicine, University of California Los Angeles, Los Angeles, California, United States of America; 9 Department of Anesthesiology, Intensive Care & Pain Management, Diakonessenhuis, Utrecht, The Netherlands; 10 Central Military Hospital, Utrecht, The Netherlands; 11 Intensive Care Unit, Vrije Universiteit Medical Center, Amsterdam, The Netherlands; 12 Department of Anesthesiology, Intensive Care & Pain Management, St Antonius Hospital, Nieuwegein, The Netherlands; 13 Division of Anesthesiology, Intensive Care and Emergency Medicine, University Medical Center Utrecht, Utrecht, The Netherlands; 14 Biostatistics School of Public Health, University of California Los Angeles, Los Angeles, California, United States of America; 15 Center for Neurobehavioral Genetics, Semel Institute for Neuroscience and Human Behavior, University of California Los Angeles, Los Angeles, California, United States of America; Morehouse School of Medicine, United States of America

## Abstract

**Background:**

Animal studies have revealed seasonal patterns in cerebrospinal fluid (CSF) monoamine (MA) turnover. In humans, no study had systematically assessed seasonal patterns in CSF MA turnover in a large set of healthy adults.

**Methodology/Principal Findings:**

Standardized amounts of CSF were prospectively collected from 223 healthy individuals undergoing spinal anesthesia for minor surgical procedures. The metabolites of serotonin (5-hydroxyindoleacetic acid, 5-HIAA), dopamine (homovanillic acid, HVA) and norepinephrine (3-methoxy-4-hydroxyphenylglycol, MPHG) were measured using high performance liquid chromatography (HPLC). Concentration measurements by sampling and birth dates were modeled using a non-linear quantile cosine function and locally weighted scatterplot smoothing (LOESS, span = 0.75). The cosine model showed a unimodal season of sampling 5-HIAA zenith in April and a nadir in October (p-value of the amplitude of the cosine = 0.00050), with predicted maximum (PC_max_) and minimum (PC_min_) concentrations of 173 and 108 nmol/L, respectively, implying a 60% increase from trough to peak. Season of birth showed a unimodal 5-HIAA zenith in May and a nadir in November (p = 0.00339; PC_max_ = 172 and PC_min_ = 126). The non-parametric LOESS showed a similar pattern to the cosine in both season of sampling and season of birth models, validating the cosine model. A final model including both sampling and birth months demonstrated that both sampling and birth seasons were independent predictors of 5-HIAA concentrations.

**Conclusion:**

In subjects without mental illness, 5-HT turnover shows circannual variation by season of sampling as well as season of birth, with peaks in spring and troughs in fall.

## Introduction

Seasonal patterns in behavior and psychiatric symptoms are present in both healthy and clinical populations. For example, in healthy humans mood is lowest in fall [1], [2], bipolar patients are at the highest risk of suffering an episode in fall [3] and suicide peaks in spring in both hemispheres [4], [5], [6], [7], [8]. Moreover, season of birth has been associated with several behavioral traits, such as smoking, novelty seeking and suicide [9], [10], [11], [12], [13]. Finally, meta-analyses have linked season of birth with schizophrenia [14], [15].

From an evolutionary perspective, seasonal adaptation of several kinds of behaviors, e.g. mating and degree of physical activity, may be advantageous. Seasonal variation in psychiatric illness may reflect such season-dependent behavioral variations in that psychiatric symptoms are at the extremes of normal behavior. Clarifying the mechanisms underlying seasonal variation in behavioral processes could thus further our understanding of the etiology of mental disorders.

Animal studies have revealed pronounced correlations between mating behavior and energy consumption (i.e. hibernation) on the one hand and serotonin (5-hydroxytryptamine, 5-HT) metabolism on the other [16], [17], [18], [19]. For example, in rhesus macaques, the main metabolite of 5-HT (5-hydroxyindoleacetic acid, 5-HIAA) in cerebrospinal fluid (CSF) is highest during the mating season and correlates positively with several measures of successful mating [17], [19]. In addition, 5-HIAA levels and monoamine oxidase (MAO)-activity increase during arousal from hibernation in ground squirrels [20]. However, knowledge about seasonal patterns in 5-HT, dopamine (DA), and norepinephrine turnover in the human brain is scant. One study describing a sample of 34 volunteers reported that concentrations of 5-HIAA and the main DA metabolite (homovanillic acid, HVA) in CSF were highest in summer [21]. Monoamine (MA) metabolite levels in the CSF of 283 newborn febrile infants showed seasonal variation [22], but it has remained unclear whether season of sampling or season of birth was the main determinant and it is unknown how this relates to MA metabolite levels in adulthood. And finally, preliminary evidence exists that in fall and winter human dopamine synthesis and storage are increased in the putamen [23].

We hypothesized that human MA turnover shows circannual fluctuations but given the inconclusiveness of the available data were unable to form any prior assumptions about their pattern. To elucidate the seasonal variation of physiological human MA turnover, we prospectively collected standardized amounts of CSF in a homogeneous sample of 223 healthy individuals undergoing spinal anesthesia for minor elective procedures and studied the effects of season of sampling and season of birth on MA metabolites.

## Results

### Subject characteristics and data completeness (Supplementary Table S1)

The characteristics of the procedures and 223 included subjects are listed in supplementary table S1. One hundred sixty-seven subjects were male (75%) and the mean age was 39 (+−11). For the two main covariates -age and sex- no data were missing. The maximum percentage of missing data for the other covariates was 5%. Seven subjects used psychotropic medication. Knee arthroscopy mostly due to meniscus injuries was by far the most common surgical procedure, comprising 77% of all operations. No correlation between storage time and metabolite concentrations or between birth and sampling dates was found.

### Concentrations of metabolites per season (Table 1)

**Table 1:** Median concentrations of monoamine metabolites (in nmol/L) in cerebrospinal fluid with standard deviations (S.D.), per season (italics indicate totals).

Season of Sampling	Number	Median	S.D
**5-HIAA**	*223*	*141*	*65*
Winter	88	139	67
Spring	53	183	67
Summer	25	139	58
Fall	57	115	49
**HVA**	*223*	*201*	*77*
Winter	88	202	82
Spring	53	200	69
Summer	25	249	71
Fall	57	181	75
**MHPG**	*223*	*24.2*	*5.4*
Winter	88	23.9	4.4
Spring	53	23.2	5.1
Summer	25	25.6	6.4
Fall	57	26.3	6.0
**Season of Birth**			
**5-HIAA**			
Winter	51	126	70
Spring	57	163	56
Summer	71	136	63
Fall	44	126	70
**HVA**			
Winter	51	174	79
Spring	57	221	85
Summer	71	187	63
Fall	44	217	77
**MHPG**			
Winter	51	24.8	5.7
Spring	57	24.0	5.0
Summer	71	24.7	5.1
Fall	44	24.2	5.4

All MA metabolite concentrations were normally distributed. Median concentrations per season are shown in table 1. Only for 5-HIAA, a clear per-season pattern of season of both sampling and birth was observed, with highest concentrations in spring and lowest in fall.

### Season of sampling (Models 1 and 2; Table 2 and Figure 1)

**Figure 1:**
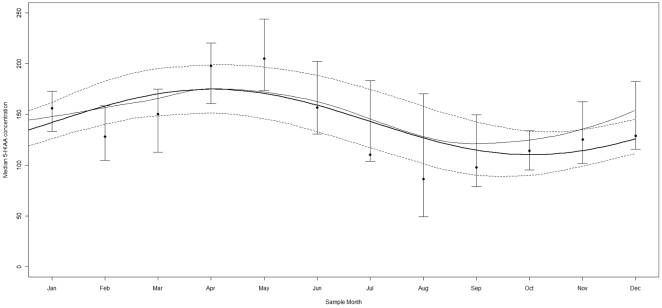
Median CSF 5-HAA Concentrations (in nmol/L) are plotted against Month of CSF Sampling. Bold line represents cosine, thin line represents LOESS and dashed lines represent 95% CIs of the cosine. Whiskers indicate 95% CIs of 5-HIAA concentrations.

**Table 2:** Amplitude of the best fitting models per metabolite with significance levels (in bold: Bonferroni-corrected significant results).

	Amplitude	p-value	t-statistic	tmax	tmin	Model
**Season of sampling**						
- 5-HIAA	**−32.26**	**0.00050**	**−3.53**	**April**	**October**	**1-peak**
- HVA	−14.95	0.09027	−1.70	Feb/August	May/Nov	2-peaks
- MHPG	1.75	0.02252	2.30	March	September	1-peak
**Season of birth**						
- 5-HIAA	**−22.70**	**0.00339**	**−2.96**	**May**	**November**	**1-peak**
- HVA	17.93	0.07157	1.81	March/Sept	June/Dec	2-peaks
- MHPG	-1.34	0.04730	-1.99	May/Nov	August/Feb	2-peaks

T-statistic of the amplitudes, tmax (month during which levels peak), tmin (month during which levels are at their lowest), and best-fitting model based on deviance calculations. Feb = February; Nov = November; Sept = September; Dec = December.

The best fitting nlqr-model (1-peak) was significant for 5-HIAA (Figure 1 and table 2): the A (amplitude) was −32.3, p = 0.00050; t_max_ = 4.04 (April) and t_min_ 10.0 (October). HVA and MHPG amplitude results were non-significant (table 2).

The predicted concentrations at t_max_ (PC_max_) and at t_min_(PC_min_) for 5-HIAA were: 173 and 108 nmol/L, respectively, implying a 60% increase from October to April. Deviances and β's of the nlqr-models are given in supplementary table S2.

The LOESS for 5-HIAA followed a similar pattern to the cosine function (figure 1, Kruskal-Wallis p-value of the three peak months that turned out to be sequential –April, May, June- vs the other months = 3.90×10^−6^), validating the chosen cosine model. Supplementary figure S1 provides the raw data points with cosine and LOESS lines.

### Season of birth (Models 1 and 2; Table 2 and Figure 2)

**Figure 2:**
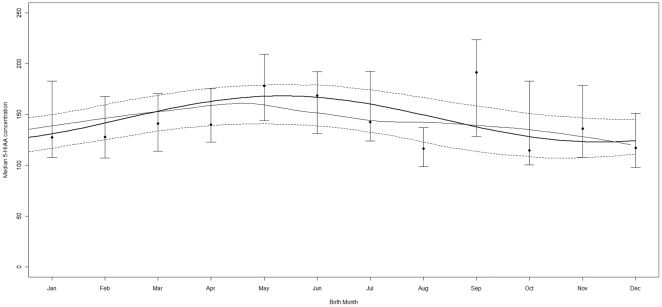
Median CSF 5-HAA Concentrations (in nmol/L) are plotted against Month of Birth. Bold line represents cosine, thin line represents LOESS and dashed lines represent 95% CIs of the cosine. Whiskers indicate 95% CIs of 5-HIAA concentrations.

The best fitting model (1-peak) was significant for 5-HIAA (Figure 2 and table 2): the A was −22.7 (p = 0.00339); t_max_ = 5.34 (May) and t_min_ = 11.3 (November). Amplitudes for the other MA metabolites were non-significant (table 2).

The predicted concentrations at t_max_ (PC_max_) and at t_min_ (PC_min_) for 5-HIAA were: 172 and 126 nmol/L, respectively, implying a 37% higher concentration for those born in May compared to November. Deviances and β's of the nlqr-models are given in supplementary table S2.

The LOESS for 5-HIAA followed a similar pattern to the cosine function (figure 2, no Kruskal-Wallis computed as the median peak months –May, June, September- were not sequential), suggesting our cosine modeling approach was also valid for the season of birth 5-HIAA analysis. Supplementary figure S2 provides the raw data points with cosine and LOESS lines.

### Sampling and birth within one model (Model 3; supplementary table S2)

For all MA metabolites, model 3 improved the goodness-of-fit compared to the sampling and birth models 1 and 2. The 5-HIAA one-peak model 3 showed similarly significant sampling (p = 0.00017) and birth (p = 0.00756) amplitudes to model 1. In addition, the peaks and troughs of birth and sampling remained similar (sampling PC_max_ = 3.75 and PC_min_ = 9.75; birth PC_max_ = 5.40 and PC_min_ = 11.4). The amplitude significances of the HVA two-peak model 3 were similarly non-significant to model 2. For MHPG, the one-peak model 3 showed a similarly non-significant birth amplitude to model 2, but a significant sampling amplitude (p = 0.00589, in contrast to p = 0.02252 in model 1; PC_max_ = 3.61 and PC_min_ = 9.61).

## Discussion

In the first study on this topic in healthy human participants (n = 223), all three main monoamine metabolites in CSF were modeled by sampling and birth dates using a non-linear cosine function and LOESS (locally weighted scatterplot smoothing). Sampling and birth in spring were associated with unimodal peaks in 5-HIAA concentrations.

### Hypothesized functions and mechanism of high spring 5-HIAA

Conception is the most seasonally driven behavior across species [24] and peaks in spring in most species, including humans [25]. Evidence supporting a role for 5-HT in mating behavior of animals abounds. CSF 5-HIAA is highest during the mating season and correlates positively with sexual competence in rhesus macaques [17], [19]. In addition, catfish have high levels of monoamine-oxidase (MAO) activity before reproduction whereas the inverse applies to the spawning (reproduction) phase [16]. MAO is the main 5-HT breakdown enzyme and increased MAO levels may consequently correspond with high 5-HT turnover (reflected by the 5-HIAA/5-HT-ratio). Provided in humans 5-HIAA concentrations also positively correlate with successful reproductive behavior, early spring would thus be the most appropriate time of year for 5-HIAA peaks, increasing the likelihood of spring or summer births. Such a hypothesis is in keeping with our findings of highest 5-HIAA concentrations in early spring.

**Figure 3:**
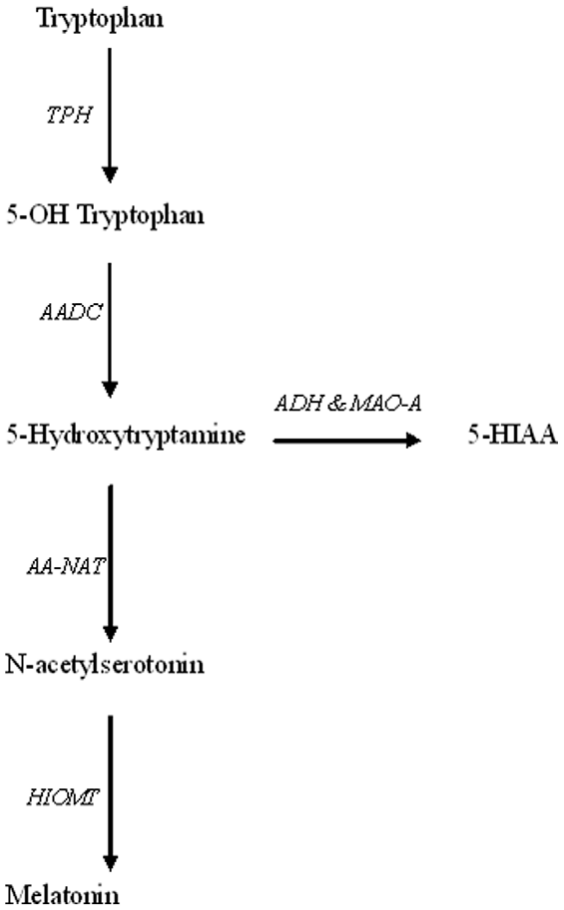
Metabolism of 5-hydroxytryptamine (5-HT, serotonin). Enzymes in italics. *TPH = tryptophan hydroxylase; AADC = aromatic amino acid decarboxylase; ADH = alcohol dehydrogenase; MAO = monoamine oxidase*; 5-HIAA = 5-hydroxyindoleacetic acid; *AA-NAT = arylalkylamine N-acetyltransferase; HIOMT = hydroxyindole-O-methyltransferase*.

One plausible mechanism underlying our finding of highest 5-HIAA in early spring relates to differences in light exposure throughout the year. Circannual melatonin (5-methoxy-N-acetyltryptamine) and 5-HT up and down regulation are likely to be interdependent, as 5-HT is the precursor of melatonin (figure 3). Melatonin is synthesized within pinealocytes from 5-HT by two enzymes – arylalkylamine N-acetyltransferase (AA-NAT) and hydroxyindole-*O*-methyltransferase (HIOMT) [26]. Its functions range from circadian (most importantly sleep cuing) to circannual (melatonin as a seasonal zeitgeber for reproduction and puberty) across a wide range of species [24], [27]. In humans, only preliminary evidence indicates that pinealectomized subjects are less seasonal than individuals with an intact pineal gland [28], which may be related to blunting of melatonin peaks due to the abundance of artificial light in modern society [27]. In the pineal gland of the European hamster, however, the activity and gene expression of AA-NAT are three to eight times increased in November compared to June [26]. This is in accordance with the higher basal and nighttime melatonin concentrations in fall compared to spring that have been demonstrated for several species [26], [27]. Linking this knowledge about seasonal variation in the melatonin pathway to our findings leads us to posit that more 5-HT is used by the human body to produce melatonin in fall than in spring. This hypothesis would explain two phenomena. First, more 5-HT may be available for other purposes –such as conception- in spring than in fall. And second, the 5-HIAA/5-HT-ratio would be higher in spring than in fall, which is in keeping with the high concentrations of 5-HIAA we detected in spring.

### Season of birth effects

Our finding of increased CSF 5-HIAA levels in those born in spring supports available data showing a primary role of 5-HT turnover in fitness and survival. That especially 5-HIAA concentrations in CSF (and to a lesser degree HVA and MHPG) are stable from childhood into adulthood, has been demonstrated in rhesus macaques [29]. Moreover, high 5-HIAA levels are associated with an increased likelihood of survival in these primates [29], [30]. Until recently in human history, early spring was an auspicious period to start the reproduction cycle as the likelihood of a child being born in spring is then also increased. Such reasoning is supported by epidemiological data showing that European women are most likely to become pregnant within six months and only have an approximately 20% chance of becoming pregnant after one month [31]. In fact, a Finnish study demonstrated that births peaked in early spring from 1650 through 1850 [32]. Although no data are currently available to test such a correlation in humans, it is thus conceivable that in the pre-industrialization era high CSF 5-HIAA concentrations in spring-born humans advanced adaptability and survival, which is supported by our data. On the other hand, other studies have found high suicide rates among spring and summer-born individuals {Dome, 2010 #82}{Salib, 2006 #109}. Although speculative as no empirical data are currently available to test such a hypothesis, one may reason that a difference in monoamine turnover seasonality between suicide committers and healthy subjects (our study population) is one of the determinants of suicide predisposition. Those born in summer and spring may thus only be at risk to commit suicide if their 5-HIAA peak is attenuated compared to healthy subjects.

### Neurobehavioral disorders

How may our findings be viewed in light of studies showing highest levels of 5-HIAA in other seasons than spring in neuropsychiatric patients? Differences in seasonal variation between cases and controls may partly explain such apparent disagreement, in that patients may display either an attenuated or an opposite pattern of seasonal variation in MA metabolism. Two studies provide preliminary evidence for the former; one in a sample of children suffering from a variety of psychiatric disorders (n = 72) and the other in alcohol dependent patients (n = 135). In neither of the two reports seasonal differences in monoamine metabolites were detected [33], [34]. Similarly, given previously detected peaks of MA metabolite levels in winter born psychiatric patients [35], the influence of season of birth on monoamine metabolite levels seems to differ between patients and healthy controls. In this context, a prime disorder of interest is seasonal affective disorder (SAD), in which the prominent characteristic is seasonal variation in semiology. Interestingly, also its pathophysiology is seasonally determined as melatonin rhythms in SAD patients are delayed compared to controls [36], [37], which in turn may be related to seasonal shifts in the 5-HT-melatonin pathway.

Suicide is another season-associated psychiatric phenotype, for which several studies support an early spring incidence zenith [4], [5], [6], [7], [8]. It is conceivable that seasonal variation in the foremost neurotransmitter system associated with suicide -5-HT- plays a role in the pathophysiology of season-associated suicide. As suicide is most frequent among severe psychiatric disorders with a chronic course and we have not included such patients in our study population, possible differences in seasonal variation of 5-HT turnover between such patients and healthy individuals remain hypothetical.

### Limitations and future directions

To our knowledge, no previous study on seasonal variation in any animal or human metabolite to date incorporated an assumption-free LOESS model into a cosine (that includes both season of sampling and season of birth) after systematically correcting for all possibly relevant covariates. This statistical approach has made our findings robust to type I and II errors, although a methodological concern may be selection bias as only patients undergoing minor elective procedures were included. On the other hand, available epidemiological data obtained from over 400,000 knee arthroscopies (by far the most common procedure in this study) suggest that such patients reflect the general population, for example with regard to history of cancer and comorbidities [38]. In addition, the standardized sampling conditions in our operating rooms are likely to have benefitted the reliability of intersubject MA metabolite concentration comparisons.

To detect possible gender-dependent differences in seasonal patterns, we ran the 1-peak 5-HIAA models for men and women separately. Seasonal variation in both season of birth and season of sampling remained similar with all models yielding Bonferroni-corrected significant amplitudes and similar t_max_ and t_min_, with the exception of season of birth where the same peaks and troughs were visible but significance decreased to 0.042 for women and 0.046 for men. Excluding the 7 subjects on psychotropic medication from our analyses also resulted in similar findings and significance levels (supplementary table S2).

Limiting the interpretability of our season of birth findings are the moderate significance of the season of birth nlqr model and the unexpectedly high September concentrations. A limitation of our method is that MA metabolites at one point in time per person have been assessed. Future longitudinal studies over several years in the same study population and studies comparing healthy subjects with neuropsychiatric patients may fill the current lacunae in our understanding of seasonal variability in monoamine turnover. In addition, based on our data the potential effects of MAO-A activity cannot be teased apart, as high 5-HIAA concentrations may either point to increased breakdown or to an altogether upregulated 5-HT system, including more biosynthesis. Although we cannot draw firm conclusions about causal directions based on our findings, animal studies hint at 5-HT up-regulation in spring [39]. In vivo assessments of MAO-A activity (e.g. by PET [40] and gene expression profiling) in subjects with measured CSF 5-HIAA-concentrations may thus provide new insights into the seasonality of human 5-HT physiology.

## Materials and Methods

### Subjects

This study was approved by the ethics committee at the University Medical Center Utrecht (UMCU) and all local ethics committees. Volunteers were recruited at outpatient pre-operative screening services in four hospitals in and around Utrecht, The Netherlands, from August 2008 until March 2010: UMCU, Central Military Hospital, Sint Antonius Hospital, and Diakonessenziekenhuis. We included patients (i) undergoing spinal anaesthesia for minor elective surgical procedures, (ii) ranging between 18–60 years of age, and (iii) with four grandparents born in The Netherlands or other North-Western European countries (Belgium, Germany, UK, France, and Denmark). Written informed consent was obtained from all participants. Subjects suffering current or past major psychiatric or neurological disorders were excluded during a telephone interview. During this interview J.L. (a psychiatry resident) or a medical student trained by J.L, excluded subjects who reported they currently or in the past (had) suffered any psychiatric or neurological illness or had been admitted to a psychiatric or neurological unit (n = 5).

### Collection of CSF

Subjects had fasted at least 6 hours prior to lumbar puncture (LP). Before administration of medication (either pre-medication or compounds for the purpose of anesthesia), a 25–27 Gauge needle was inserted into the L1/L2, L2/L3, L3/L4, or L4/L5 interspace (estimated by the anaesthesiologist). A single sample of 6 mL of CSF was obtained from each subject. Age, height, weight, time of procedure, duration of aspiration (usually 30–60 seconds), type of procedure, and diagnosis related to the procedure were recorded. In addition, any deviations from the instructed procedure were recorded, such as smaller amounts of CSF drawn or operation complications. CSF was kept at 4°C and transported within 5 hours to the laboratory at UMCU. Each sample was immediately stored in fractions of 0.5 mL and 1 mL at −80°C. One fraction of 0.5 mL was used for MA metabolite measurements.

### Monoamine metabolite measurements

Concentrations of CSF MA metabolite levels (3-methoxy-4-hydroxyphenylglycol, MHPG; 5-HIAA; and HVA) were measured using high performance liquid chromatography (HPLC; Dionex-Thermo Fisher, USA) with electrochemical detection (DecadeII, Antec, Leiden, the Netherlands). The CSF samples were thawed, mixed and centrifuged, after which 50 µL was injected on a reverse phase C18 (150×4.6 mm, 3 µm) HPLC column (Supelco, Sigma-Aldrich). The MA metabolites were eluted with a mixture of methanol (8% final concentration) and a phosphate buffer (50 mM, pH3) containing 0.2 g/L octane-sulphonic acid and 0.2 g/L Na-EDTA. Separate stock standards of MHPG, 5-HIAA and HVA (Sigma, St Louis, USA) were prepared in a 0.2 mol/L HCL-154 mmol/L NaCL solution at concentrations of 502, 506 and 502 µmol/L, respectively. A mixture of the working external standards was made by diluting the stock solutions in 154 mmol/L NaCL to a final concentration of 100, 502 and 1012 nmol/L, respectively. In-house quality controls were prepared by mixing separate stock solutions of the MA metabolites to final concentrations of 500 nmol/L for HVA and 5-HIAA and 50 nmol/L for MHPG. All MA metabolites in each sample were measured twice. Peak areas were measured and compared to those of the external standards for quantitation. The electochemical detector settings of the conditioning and analytical cells were +150 mV +400 mV, respectively. Samples showing >10% differences (15% for MHPG) between the first and second measurement were measured again until the difference was <10% (which applied to a total of <5% of all measurements). For each sample, the mean of the two measurements was used for further analyses. Day-to-day imprecision (for single measurements) ranged from 4 to 10% for 5-HIAA, from 5 to 10% for HVA and 4 to 8% for MHPG.

### Statistical analyses

MA metabolite concentration distributions were verified for normality using the Kolmogorov-Smirnov test (p-values >0.05 were considered indicative of a normal distribution). Median concentrations per season (defined as starting on the 21^st^ and ending on the 20^th^ three months later, e.g. spring starting March 21^st^ and ending June 20^th^) were computed. Non-linear quantile regression (nlqr) – which models the median values per month - was chosen before inspecting circannual metabolite concentration variability to evaluate the effects of season of sampling and season of birth on MA metabolite concentrations assuming a cosine-shaped relationship. (Median values were used because of uneven distributions of sampling and birth dates). To validate the cosine function and model concentration measurements per day non-parametrically, we used LOESS (locally weighted scatterplot smoothing), which offers the advantage of modeling data points without having to set a function according to which the data are described. The default span in R 2.12.1 (www.r-project.org) of 0.75 was used. In the event three months with highest median MA metabolite concentrations were sequential within the same season, the non-parametric group comparison Kruskal-Wallis test was used to compare metabolite levels between that season and the other seasons taken together. Kruskal-Wallis test results with p-values<0.05 were deemed significant. Two covariates, age and sex, were included in all nlqr-models. Other potentially confounding factors (amount of CSF suctioned, type and timing of procedure, comorbidities (psychiatric and other), psychotropic medication and other medication, LP level, and height and weight of participants) were investigated for association with MA metabolites by means of univariate linear regression after multiple (n = 5) imputation of missing values in SPSS 18.0 (SPSS for Windows, SPSS Inc). Factors that showed a univariate association (p<0.05) and did not show collinearity with age or sex (Pearson's r<0.6) were additionally entered into the models (which was the case for LP level and weight in the HVA model). Using Pearson's r, it was checked whether storage time and metabolite concentrations and whether sampling and birth date correlated. Since we hypothesized circannual variation with either one or two zeniths and nadirs per year would apply to all three MA metabolites, nlqr-models were fitted for each of these two scenarios. We measured the fit of each model per MA metabolite and compared the deviances (residual sum of squares). Thus, two nlqr-models were compared for goodness-of-fit for each of the six analyses (season of sampling and season of birth being the predictors for each of the three MA metabolites) and only results for the best fitting model were reported. To determine whether birth and sampling dates independently contributed to MA metabolite concentrations, a third model including both sampling and birth months within one model was created.

**Model 1a (5-HIAA and MHPG, one peak):**

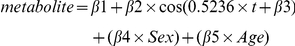



**Model 1b (5-HIAA and MHPG, two peaks):**

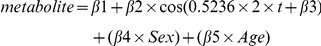



**Model 2a (HVA, one peak):**

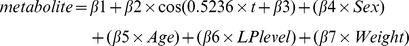



**Model 2b (HVA, two peaks):**

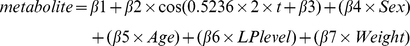



**Model 3a (5-HIAA and MHPG, sampling and birth month within one model, one peak):**

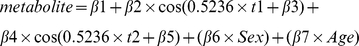



**Model 3b (5-HIAA and MHPG, sampling and birth month within one model, two peaks):**

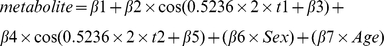



**Model 3c (HVA, sampling and birth month within one model, one peak):**

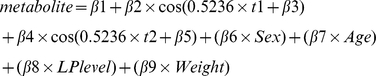



**Model 3d (HVA, sampling and birth month within one model, two peaks):**

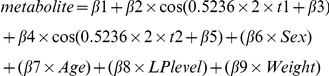



In which:

metabolite = concentration of MA metabolite
β1 = baseline level
β2 = amplitude (A)
0.5236 = coefficient of t = 2π/12 (one cosine period in radians divided by the number of months per year; “x 2” added for the two-peaks model)
t = month of sampling or birth; t1 = month of sampling; t2 = birth month
β3 = phase shift
β4, β5, β6, β7, β8, and β9 = covariates' coefficients (except for in model 3, where β4 and β5 are amplitude and phase shift, respectively)

For each 1-peak model showing a significant amplitude the t_max_ (month during which a level is at its maximum), the t_min_ (month during which a level is at its minimum), and predicted maximum (PC_max_) and minimum (PC_min_) concentrations were computed:
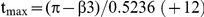
and the predicted concentration at t_max_:
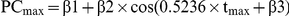


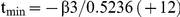
and the predicted concentration at t_min_:
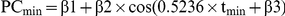



In each of the best fitting models, the effect of season of sampling or season of birth was considered significant when the amplitude (A) was significantly different from zero. The nlqr significance level was Bonferroni corrected and set at 0.05/6 = 0.00833 (since three tests for both season of sampling and season of birth were performed). All data analyses were performed with the statistical software package SPSS 18.0 (SPSS for Windows, SPSS Inc) and R 2.12.1 (www.r-project.org).

## Supporting Information

Figure S1 Raw values of CSF 5-HAA Concentrations (in nmol/L) are plotted against Month of CSF Sampling. Bold line represents cosine, thin line represents LOESS and dashed lines represent 95% CIs of the cosine.
(TIFF)

Figure S2 Raw values of CSF 5-HAA Concentrations (in nmol/L) are plotted against Month of Birth. Bold line represents cosine, thin line represents LOESS and dashed lines represent 95% CIs of the cosine.
(TIFF)

Table S1 Subject characteristics; means are given for all variables except for sex and procedure type (for which absolute numbers and percentages are shown).
(DOC)

Table S2 Deviances and results of each model per monoamine metabolite.
(DOC)

## References

[1] Harris S, Dawson-Hughes B (1993). Seasonal mood changes in 250 normal women.. Psychiatry Res.

[2] Chotai J, Smedh K, Johansson C, Nilsson LG, Adolfsson R (2004). An epidemiological study on gender differences in self-reported seasonal changes in mood and behaviour in a general population of northern Sweden.. Nord J Psychiatry.

[3] Friedman E, Gyulai L, Bhargava M, Landen M, Wisniewski S (2006). Seasonal changes in clinical status in bipolar disorder: a prospective study in 1000 STEP-BD patients.. Acta Psychiatr Scand.

[4] Bjorksten KS, Bjerregaard P, Kripke DF (2005). Suicides in the midnight sun–a study of seasonality in suicides in West Greenland.. Psychiatry Res.

[5] Partonen T, Haukka J, Viilo K, Hakko H, Pirkola S (2004). Cyclic time patterns of death from suicide in northern Finland.. J Affect Disord.

[6] Chew KS, McCleary R (1995). The spring peak in suicides: a cross-national analysis.. Soc Sci Med.

[7] Rocchi MB, Perlini C (2002). Is the time of suicide a random choice? A new statistical perspective.. Crisis.

[8] Heerlein A, Valeria C, Medina B (2006). Seasonal variation in suicidal deaths in Chile: its relationship to latitude.. Psychopathology.

[9] Riala K, Hakko H, Taanila A, Rasanen P (2009). Season of birth and smoking: findings from the Northern Finland 1966 Birth Cohort.. Chronobiol Int.

[10] Chotai J, Forsgren T, Nilsson LG, Adolfsson R (2001). Season of birth variations in the temperament and character inventory of personality in a general population.. Neuropsychobiology.

[11] Eisenberg DT, Campbell B, Mackillop J, Lum JK, Wilson DS (2007). Season of birth and dopamine receptor gene associations with impulsivity, sensation seeking and reproductive behaviors.. PLoS One.

[12] Dome P, Kapitany B, Ignits G, Rihmer Z (2010). Season of birth is significantly associated with the risk of completed suicide.. Biol Psychiatry.

[13] Chotai J, Joukamaa M, Taanila A, Lichtermann D, Miettunen J (2009). Novelty seeking among adult women is lower for the winter borns compared to the summer borns: replication in a large Finnish birth cohort.. Compr Psychiatry.

[14] Davies G, Welham J, Chant D, Torrey EF, McGrath J (2003). A systematic review and meta-analysis of Northern Hemisphere season of birth studies in schizophrenia.. Schizophr Bull.

[15] Messias E, Kirkpatrick B, Bromet E, Ross D, Buchanan RW (2004). Summer birth and deficit schizophrenia: a pooled analysis from 6 countries.. Arch Gen Psychiatry.

[16] Manickam P, Joy KP (1989). Changes in hypothalamic monoamine oxidase activity in relation to season, ovariectomy, and 17 beta-estradiol administration in intact and ovariectomized catfish, Clarias batrachus (L.).. Gen Comp Endocrinol.

[17] Mehlman PT, Higley JD, Fernald BJ, Sallee FR, Suomi SJ (1997). CSF 5-HIAA, testosterone, and sociosexual behaviors in free-ranging male rhesus macaques in the mating season.. Psychiatry Res.

[18] Murakami N, Kono R, Nakahara K, Ida T, Kuroda H (2000). Induction of unseasonable hibernation and involvement of serotonin in entrance into and maintenance of its hibernation of chipmunks T. asiaticus.. J Vet Med Sci.

[19] Zajicek KB, Price CS, Shoaf SE, Mehlman PT, Suomi SJ (2000). Seasonal variation in CSF 5-HIAA concentrations in male rhesus macaques.. Neuropsychopharmacology.

[20] Popova NK, Voitenko NN (1981). Brain serotonin metabolism in hibernation.. Pharmacol Biochem Behav.

[21] Brewerton TD, Berrettini WH, Nurnberger JI, Linnoila M (1988). Analysis of seasonal fluctuations of CSF monoamine metabolites and neuropeptides in normal controls: findings with 5HIAA and HVA.. Psychiatry Res.

[22] Chotai J, Murphy DL, Constantino JN (2006). Cerebrospinal fluid monoamine metabolite levels in human newborn infants born in winter differ from those born in summer.. Psychiatry Res.

[23] Eisenberg DP, Kohn PD, Baller EB, Bronstein JA, Masdeu JC (2010). Seasonal effects on human striatal presynaptic dopamine synthesis.. J Neurosci.

[24] Arendt J The Pineal Gland and Pineal Tumours - NEUROENDOCRINOLOGY, HYPOTHALAMUS, and PITUITARY.. http://WWW.ENDOTEXT.ORG.

[25] Roenneberg T, Aschoff J (1990). Annual rhythm of human reproduction: I. Biology, sociology, or both?. J Biol Rhythms.

[26] Garidou ML, Vivien-Roels B, Pevet P, Miguez J, Simonneaux V (2003). Mechanisms regulating the marked seasonal variation in melatonin synthesis in the European hamster pineal gland.. Am J Physiol Regul Integr Comp Physiol.

[27] Macchi MM, Bruce JN (2004). Human pineal physiology and functional significance of melatonin.. Front Neuroendocrinol.

[28] Macchi MM, Bruce JA, Boulos Z (2002). Sleep, chonotype and seasonality after pineal resection in humans: inital findings.. Society for Research on Biological Rhythms Abstracts.

[29] Howell S, Westergaard G, Hoos B, Chavanne TJ, Shoaf SE (2007). Serotonergic influences on life-history outcomes in free-ranging male rhesus macaques.. Am J Primatol.

[30] Higley JD, Mehlman PT, Higley SB, Fernald B, Vickers J (1996). Excessive mortality in young free-ranging male nonhuman primates with low cerebrospinal fluid 5-hydroxyindoleacetic acid concentrations.. Arch Gen Psychiatry.

[31] Juul S, Karmaus W, Olsen J (1999). Regional differences in waiting time to pregnancy: pregnancy-based surveys from Denmark, France, Germany, Italy and Sweden. The European Infertility and Subfecundity Study Group.. Hum Reprod.

[32] Fellman J, Eriksson AW (2009). Temporal and regional variations in the seasonality of births in Aland (Finland), 1653–1950.. Biodemography Soc Biol.

[33] Swedo SE, Kruesi MJ, Leonard HL, Hamburger SD, Cheslow DL (1989). Lack of seasonal variation in pediatric lumbar cerebrospinal fluid neurotransmitter metabolite concentrations.. Acta Psychiatr Scand.

[34] Roy A, Adinoff B, DeJong J, Linnoila M (1991). Cerebrospinal fluid variables among alcoholics lack seasonal variation.. Acta Psychiatr Scand.

[35] Chotai J, Asberg M (1999). Variations in CSF monoamine metabolites according to the season of birth.. Neuropsychobiology.

[36] Lewy AJ, Sack RL, Miller LS, Hoban TM (1987). Antidepressant and circadian phase-shifting effects of light.. Science.

[37] Sack RL, Lewy AJ, White DM, Singer CM, Fireman MJ (1990). Morning vs evening light treatment for winter depression. Evidence that the therapeutic effects of light are mediated by circadian phase shifts.. Arch Gen Psychiatry.

[38] Hetsroni I, Lyman S, Do H, Mann G, Marx RG (2011). Symptomatic pulmonary embolism after outpatient arthroscopic procedures of the knee: the incidence and risk factors in 418,323 arthroscopies.. J Bone Joint Surg Br.

[39] Nagayama H, Lu JQ (1998). Circadian and circannual rhythms in the function of central 5-HT1A receptors in laboratory rats.. Psychopharmacology (Berl).

[40] Fowler JS, Alia-Klein N, Kriplani A, Logan J, Williams B (2007). Evidence that brain MAO A activity does not correspond to MAO A genotype in healthy male subjects.. Biol Psychiatry.

