# Supplementary figures and images for: Season of Sampling and Season of Birth Influence Serotonin Metabolite Levels in Human Cerebrospinal Fluid
Source: PLoS One. 2012 Feb 1;7(2):e30497. doi: 10.1371/journal.pone.0030497 (PMC3270010; doi:10.1371/journal.pone.0030497)

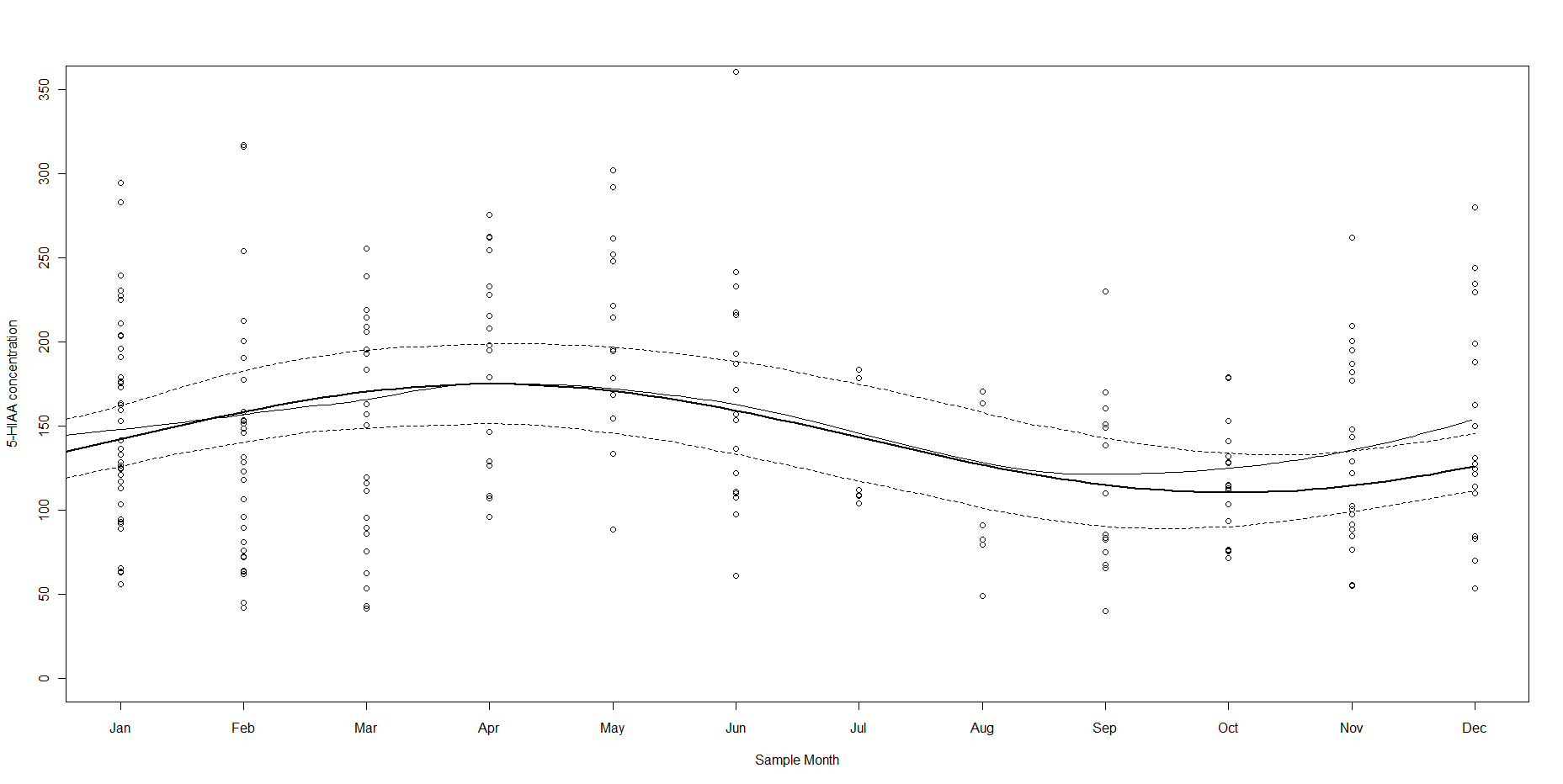

Supplement: Figure S1 — Raw values of CSF 5-HAA Concentrations (in nmol/L) are plotted against Month of CSF Sampling. Bold line represents cosine, thin line represents LOESS and dashed lines represent 95% CIs of the cosine. (TIFF) [file pone.0030497.s001.tiff]

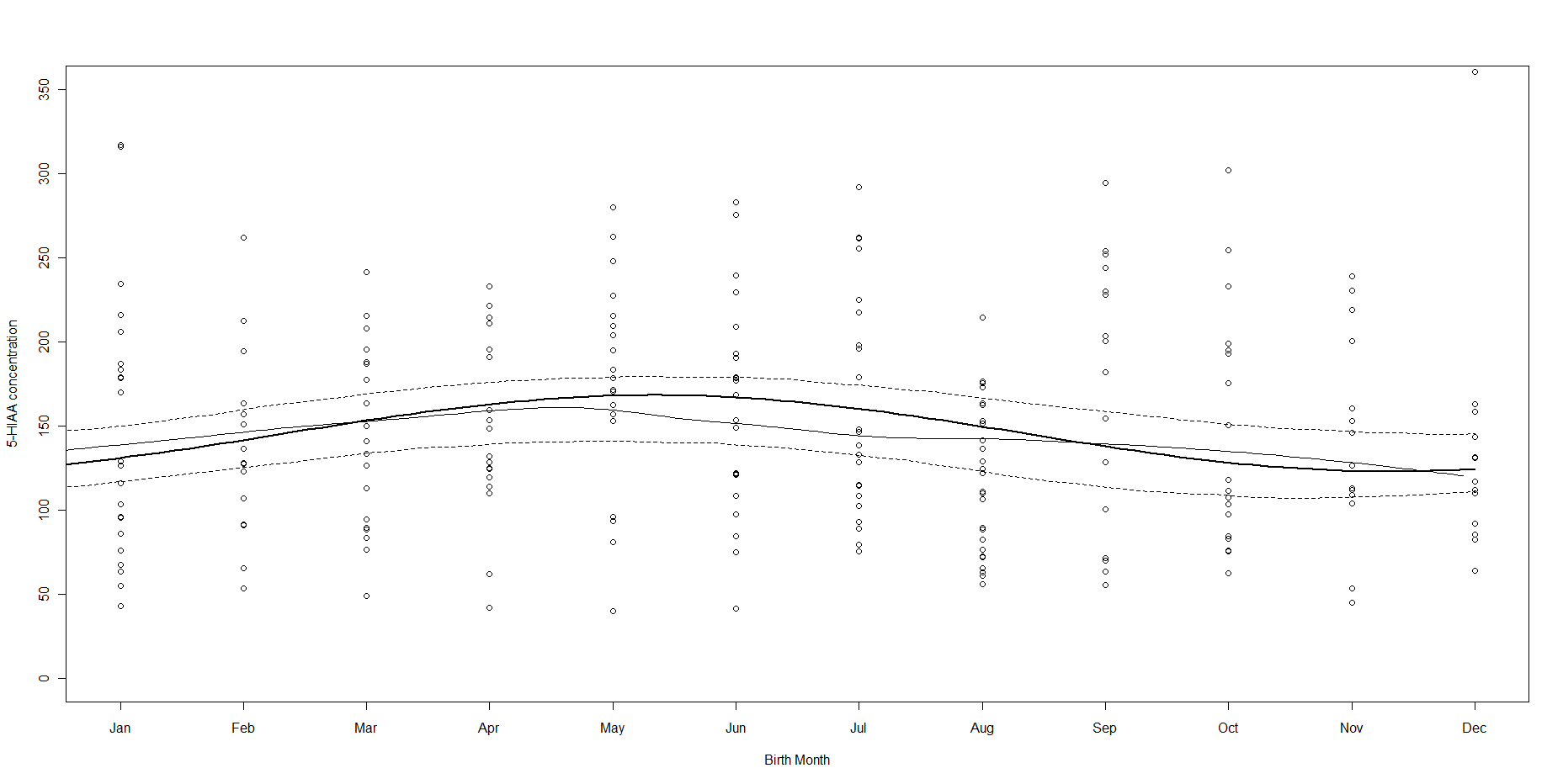

Supplement: Figure S2 — Raw values of CSF 5-HAA Concentrations (in nmol/L) are plotted against Month of Birth. Bold line represents cosine, thin line represents LOESS and dashed lines represent 95% CIs of the cosine. (TIFF) [file pone.0030497.s002.tiff]
