# Supplementary material for: Season of Sampling and Season of Birth Influence Serotonin Metabolite Levels in Human Cerebrospinal Fluid
Source: PLoS One. 2012 Feb 1;7(2):e30497. doi: 10.1371/journal.pone.0030497 (PMC3270010; doi:10.1371/journal.pone.0030497)
Supplement: Table S1 — Subject characteristics; means are given for all variables except for sex and procedure type (for which absolute numbers and percentages are shown). (DOC) [file pone.0030497.s003.doc]

|  | N not missing | Mean (SD) / N (%) |
| --- | --- | --- |
| MHPG (nmol/L) | 223 | 25 (5.4) |
| 5-HIAA (nmol/L) | 223 | 149 (65) |
| HVA (nmol/L) | 223 | 211 (77) |
| Age (years) | 223 | 39 (11) |
| Sex (male) | 223 | 167 (75%) |
| Procedure type (arthroscopies) | 219 | 171 (77%) |
| Time of sampling (SD in hours) | 218 | 11.22 am (2.1) |
| Amount of CSF drawn (mL) | 223 | 5.6 (0.6) |
