# Supplementary material for: Season of Sampling and Season of Birth Influence Serotonin Metabolite Levels in Human Cerebrospinal Fluid
Source: PLoS One. 2012 Feb 1;7(2):e30497. doi: 10.1371/journal.pone.0030497 (PMC3270010; doi:10.1371/journal.pone.0030497)
Supplement: Table S2 — Deviances and results of each model per monoamine metabolite. (DOC) [file pone.0030497.s004.doc]

NLQR (non linear quantile regression) results and deviances, 223 subjects:

**NLQR-SoSampling**

**Sample Month, 5-HIAA, 1 peak**

Coefficients:

Value Std. Error t value Pr(>|t|)

beta1 140.37334 21.96840 6.38978 0.00000

beta2 -32.26330 9.13176 -3.53309 0.00050

beta3 13.59854 0.37466 36.29582 0.00000

beta4 36.72296 18.19592 2.01820 0.04480

beta5 0.29073 0.52202 0.55692 0.57815

> deviance(nlqr.model)

[1] 5472.002

> deviance(nlqr.model.2peaks)

[1] 5709.542

**C/ model with 1-peak best fit**

**Sample Month, HVA, 2 peaks**

Coefficients:

Value Std. Error t value Pr(>|t|)

beta1 311.86174 51.42601 6.06428 0.00000

beta2 -14.95758 8.78753 -1.70214 0.09027

beta3 14.30262 0.79822 17.91821 0.00000

beta4 37.22947 22.19334 1.67751 0.09499

beta5 -0.55797 0.66534 -0.83864 0.40266

beta6 -24.78646 19.30374 -1.28402 0.20060

beta7 -0.90823 0.73412 -1.23717 0.21746

> deviance(nlqr.model)

[1] 6039.395

> deviance(nlqr.model.2peaks)

[1] 6017.523

**C/ model with 2 peaks best fit**

**Sample Month, MHPG, 1 peak**

Coefficients:

Value Std. Error t value Pr(>|t|)

beta1 23.14645 1.39470 16.59595 0.00000

beta2 1.75580 0.76411 2.29785 0.02252

beta3 13.90155 0.46199 30.09045 0.00000

beta4 1.43787 1.02142 1.40772 0.16064

beta5 0.05114 0.03664 1.39551 0.16428

> deviance(nlqr.model)

[1] 459.088

> deviance(nlqr.model.2peaks)

[1] 478.0444

**C/ model with 1 peak best fit**

**NLQR-SoBirth**

**Birth Month, 5-HIAA, 1 peak**

Coefficients:

Value Std. Error t value Pr(>|t|)

beta1 149.08584 23.24557 6.41352 0.00000

beta2 -22.69691 7.66224 -2.96218 0.00339

beta3 12.91287 0.34784 37.12310 0.00000

beta4 44.69710 19.52905 2.28875 0.02305

beta5 0.19481 0.49720 0.39181 0.69558

> deviance(nlqr.model)

[1] 5634.321

>deviance(nlqr.model.2peaks)

[1] 5722.579

**C/ model with 1 peak best fit**

**Birth Month, HVA, 2 peaks**

Coefficients:

Value Std. Error t value Pr(>|t|)

beta1 289.76046 52.65656 5.50284 0.00000

beta2 17.93231 9.89987 1.81137 0.07157

beta3 13.76844 0.51949 26.50383 0.00000

beta4 39.60257 22.81432 1.73587 0.08411

beta5 -0.37704 0.67055 -0.56228 0.57455

beta6 -12.99868 18.78239 -0.69207 0.48969

beta7 -0.66938 0.70241 -0.95299 0.34174

> deviance(nlqr.model)

[1] 5948.86

>deviance(nlqr.model.2peaks)

[1] 5904.541

**C/ model with 2 peaks best fit**

**Birth Month, MHPG, 2 peaks**

Coefficients:

Value Std. Error t value Pr(>|t|)

beta1 22.95378 1.64945 13.91602 0.00000

beta2 -1.33806 0.67074 -1.99489 0.04730

beta3 13.00605 0.57778 22.51023 0.00000

beta4 0.01002 1.35959 0.00737 0.99413

beta5 0.04094 0.03831 1.06884 0.28632

> deviance(nlqr.model)

[1] 470.4027

>deviance(nlqr.model.2peaks)

[1] 464.0931

**C/ model with 2 peaks best fit**

**Model 3**

**5-HIAA, 1 peak**

Value Std. Error t value Pr(>|t|)

beta1 154.96635 20.09780 7.71061 0.00000

beta2 -34.07583 8.89316 -3.83169 0.00017

beta3 13.74034 0.25970 52.90797 0.00000

beta4 -21.27530 7.89022 -2.69641 0.00756

beta5 12.88015 0.38556 33.40657 0.00000

beta6 35.32410 14.99816 2.35523 0.01941

beta7 -0.13393 0.49181 -0.27232 0.78563

> deviance (nlrq.model)

[1] 5324.169

> deviance (nlrq.model.2peaks)

[1] 5639.053

**C/ model with 1 peak best fit**

**HVA, 2 peaks**

Value Std. Error t value Pr(>|t|)

beta1 297.38694 52.78843 5.63356 0.00000

beta2 -13.68731 9.09539 -1.50486 0.13394

beta3 13.80195 0.78728 17.53123 0.00000

beta4 19.03638 11.35135 1.67701 0.09510

beta5 13.77778 0.52990 26.00054 0.00000

beta6 45.45637 22.86559 1.98798 0.04818

beta7 -0.22070 0.64019 -0.34474 0.73065

beta8 -21.85882 19.76156 -1.10613 0.27000

beta9 -0.79587 0.71205 -1.11770 0.26503

> deviance (nlrq.model)

[1] 5944.492

> deviance (nlrq.model.2peaks)

[1] 5845.975

**C/ model with 2 peaks best fit**

**MHPG, 1 peak**

[1] 5845.975

Value Std. Error t value Pr(>|t|)

beta1 23.62364 1.58940 14.86326 0.00000

beta2 1.98563 0.71382 2.78167 0.00589

beta3 13.81373 0.39344 35.11054 0.00000

beta4 0.51445 0.76796 0.66989 0.50364

beta5 13.20567 1.32803 9.94379 0.00000

beta6 0.95632 1.09882 0.87031 0.38509

beta7 0.04230 0.04340 0.97463 0.33083

> deviance (nlrq.model)

[1] 458.2876

beta7 0.04398 0.04397 1.00005 0.31841

> deviance (nlrq.model.2peaks)

[1] 459.2431

**C/ model with 1 peak best fit**

**NLQR-7 Psychotropic Medication**

**5-HIAA, Sample Month**

Coefficients:

Value Std. Error t value Pr(>|t|)

beta1 132.85070 22.44300 5.91947 0.00000

beta2 -33.93351 10.12551 -3.35129 0.00095

beta3 13.66351 0.28781 47.47427 0.00000

beta4 38.50367 17.47157 2.20379 0.02862

beta5 0.53086 0.52053 1.01985 0.30897

**5-HIAA, Birth Month**

Coefficients:

Value Std. Error t value Pr(>|t|)

beta1 150.70661 22.84771 6.59614 0.00000

beta2 -22.54300 7.80890 -2.88683 0.00430

beta3 12.95631 0.32016 40.46760 0.00000

beta4 47.93866 17.46486 2.74486 0.00658

beta5 0.19481 0.48712 0.39991 0.68963
